# Bromhexine inhibits SARS-CoV-2 Omicron and variant pseudovirus infection via ACE2-targeted mechanisms

**DOI:** 10.3389/fphar.2025.1745277

**Published:** 2026-01-12

**Authors:** Rafael Zúñiga, Whitney Venturini, Natalia González, Paulina Valenzuela-Hormazábal, Laura Sánchez-Aros, David Ramírez, Angel Cayo, Cristian Vilos, Leandro Zúñiga

**Affiliations:** 1 Laboratorio de Fisiología Molecular, Facultad de Medicina, Universidad de Talca, Talca, Chile; 2 Departamento de Medicina Traslacional, Facultad de Medicina, Universidad Católica del Maule, Talca, Chile; 3 Departamento de Farmacología, Facultad de Ciencias Biológicas, Universidad de Concepción, Concepción, Chile; 4 Center for Nanomedicine, Diagnostic, and Drug Development (ND3), Facultad de Medicina, Universidad de Talca, Talca, Chile; 5 Center for the Development of Nanoscience and Nanotechnology (CEDENNA), Proyecto CIA250002, Facultad de Medicina, Universidad de Talca, Talca, Chile

**Keywords:** bromhexine hydrochloride, drug repurposing, omicron variant, pseudovirus, SARS-CoV-2, spike protein binding, viralentry inhibition

## Abstract

**Background:**

Severe acute respiratory syndrome coronavirus 2 (SARS‐CoV‐2) causes a highly infectious disease characterized by fever, acute respiratory illness, and pneumonia, known as coronavirus disease 2019 (COVID‐19). SARS‐CoV‐2 infects host cells through the interaction of its spike glycoprotein (S protein) with human angiotensin‐converting enzyme 2 (hACE2). Structural studies have shown that hACE2 interacts exclusively with the receptor‐binding domain (RBD) of the spike. A high binding affinity between spike and hACE2 has been linked to increased viral infection. Disrupting this interaction can reduce viral infectivity.

**Methods:**

This study aimed to assess infection using Omicron variant pseudovirus in a stable HEK‐293 cell line expressing hACE2 (HEK‐293/ACE2), treated with bromhexine hydrochloride. First, immunofluorescence and Western blot confirmed the presence of hACE2 in the stable line. Then, bromhexine concentrations for treatment were determined by cytotoxicity assays. Next, infection was evaluated using Omicron pseudoviruses carrying GFP and luciferase reporter genes. Infection levels were measured through fluorescence or luciferase activity.

**Results:**

Bromhexine reduced infection with an IC_50_ of 17.3 ± 0.9 μM. About 40% inhibition was also observed against Alpha, Beta, and Delta variants at 40 μM. Computational docking followed by molecular dynamics simulations showed that bromhexine binds to the extracellular domain of hACE2, with recurrent contacts near Phe40, Phe390, and Asn394.

**Conclusion:**

Consistent with this model, our findings support an entry‐inhibition mechanism whereby bromhexine destabilizes the SARS‐CoV‐2 spike–ACE2 interface, preventing viral entry. Overall, these results suggest bromhexine as a potential repurposing candidate and support its inclusion in therapeutic strategies aimed at both current and emerging SARS‐CoV‐2 variants.

## 1 Introduction

Severe acute respiratory syndrome coronavirus 2 (SARS-CoV-2) is an enveloped, positive-sense single-stranded RNA virus of the genus *betacoronavirus* (Andersen et al., 2020; Gorbalenya et al., 2020; Lu et al., 2020; Wang C. et al., 2020) that belongs to the *Coronaviridae* family. It is the causative agent of the highly infectious disease characterized by fever, acute respiratory illness, and pneumonia, the coronavirus disease 2019 (COVID-19) (Gorbalenya et al., 2020; Zhu et al., 2020). SARS-CoV-2 is the third highly transmissible and pathogenic coronavirus to appear in humans, characterized by a high mortality rate.

The SARS-CoV-2 genome, which is about 30 kilobases (kb) long (Lu et al., 2020), encodes for 16 non-structural proteins, 4 structural proteins, and several accessory proteins (Stadler et al., 2003; Zhou et al., 2019; Yang and Rao, 2021; Hardenbrook and Zhang, 2022). The coronavirus structural proteins that form the viral particle include the nucleocapsid (N), membrane (M), envelope (E), and spike (S) proteins (Chan et al., 2020; Chen et al., 2020; Wu et al., 2020). These proteins are less conserved than non-structural proteins and are essential for the viral life cycle (Gil et al., 2020). The spike glycoprotein (S protein), which consists of about 1,200 to 1,400 amino acid residues per monomer (Gil et al., 2020), assembles as a homotrimer and is embedded in multiple copies within the viral envelope (Jackson et al., 2022). The trimeric S glycoprotein of SARS-CoV-2 is a class I viral fusion protein (Bosch et al., 2003) cleaved into S1 and S2 subunits by proteases such as furin during its biosynthesis in the infected cells (Hoffmann et al., 2020a; Shang et al., 2020). On the mature virion, the S protein consists of two non-covalently linked subunits: the S1 subunit, which binds to the host cell receptor, and the S2 subunit, which anchors the S protein to the membrane (Walls et al., 2020).

The S1 subunit contains four domains: the amino-terminal (N-terminal) domain (NTD), the receptor-binding domain (RBD) or S_B_, and two carboxy-terminal (C-terminal) domains (CTD1 and CTD2) (Jackson et al., 2022). The three RBDs of the S trimer form the apex of the S protein, which adopts two distinct conformations: “up” for a receptor-accessible state and “down” for a receptor-inaccessible state (Wrapp et al., 2020; Zhou T. et al., 2020). The S2 subunit also includes a fusion peptide (FP), heptad repeat 1 (HR1), heptad repeat 2 (HR2), transmembrane (TM), and cytoplasmic tail (CT) (Wang M.-Y. et al., 2020; Wrapp et al., 2020; Dutta and Acharya, 2024), all of which are necessary to mediate membrane fusion during host cell entry (Fehr and Perlman, 2015).

Recognition and binding to the host cell receptor are the initial steps of how the virus enters the host cell, leading to infection (Dutta and Acharya, 2024). The SARS-CoV-2 virus infects host cells by interacting with its surface trimeric S protein and the primary virus receptor, human angiotensin-converting enzyme 2 (hACE2) (Li et al., 2003; Hoffmann et al., 2020b; Zhou P. et al., 2020; Xiao et al., 2021). Although the respiratory tract is the main route of infection for SARS-CoV-2, the highest levels of ACE2 expression are observed in the small intestine, testis, kidneys, heart muscle, colon, and thyroid gland (Wang Y. et al., 2020; Jackson et al., 2022).

Human ACE2 (hACE2) is a type I transmembrane glycoprotein expressed in the lungs, heart, kidneys, and intestines (Donoghue et al., 2000; Yan et al., 2020). Structurally, hACE2 forms a dimer (Yan et al., 2020) and has an extracellular ectodomain with metallopeptidase activity (Tikellis et al., 2011; Xiao et al., 2021). This membrane-bound carboxypeptidase acts as a crucial negative regulator of the renin–angiotensin system (RAS), which modulates vascular function (Tikellis et al., 2011).

Cryo-EM structural studies have shown that hACE2 can only bind to one RBD (receptor-binding domain) of the SARS-CoV-2 S protein when it is in an up conformation, making it receptor-accessible (Cai et al., 2020; Wrapp et al., 2020; Zhou T. et al., 2020; Xiao et al., 2021). A significant binding affinity between the viral S protein and the host cell’s hACE2 receptor has been shown to increase viral infectivity (Hoffmann et al., 2020b; Wang Q. et al., 2020; Wrapp et al., 2020). Preventing the entry of coronaviruses into host cells before their replication and extensive mutation remains one of the most promising therapeutic strategies with significant clinical value, especially when new viruses are detected and effective treatments or vaccines are unavailable. Therefore, disrupting this molecular interaction will decrease viral infectivity. Analysis of the SARS-CoV-2 human interactome has shown that several drugs—such as disulfiram, auranofin, gefitinib, suloctidil, and bromhexine—could serve as potential COVID-19 treatments because of their different mechanisms of action (Alegría-Arcos et al., 2022). The mucolytic agent, bromhexine, a transmembrane serine protease 2 (TMPRSS2) inhibitor, has also been proposed for COVID-19 therapy (Gil et al., 2020).

Given the urgent need for antiviral strategies that target the early stages of SARS-CoV-2 infection, in this study we explored whether the drug bromhexine could interfere with viral entry processes. To address this, we employed a pseudovirus system—a replication-deficient viral model engineered to express the SARS-CoV-2 spike protein—aimed to assess infection efficiency using the Omicron variant pseudovirus in a stable HEK-293 cell line expressing human ACE2 (HEK293/ACE2), in the presence of bromhexine hydrochloride. The pseudovirus system allows studying viral entry without the risks associated with handling live viruses. First, immunofluorescence and Western blot confirmed the presence of hACE2 in the stable line. Next, bromhexine concentrations for treatment were determined using cytotoxicity assays. The effect of infection was then assessed with Omicron pseudoviruses engineered with reporter genes GFP and luciferase. Infection levels were measured by detecting fluorescence and luciferase activity. Additionally, the effectiveness was also tested against different pseudovirus variants (Alpha, Beta, and Gamma).

Furthermore, a molecular analysis was performed to verify the interaction of bromhexine with the Omicron spike and the human hACE-2 receptor. The analysis identified hACE2 residues Phe40, Phe390, and Asn394 as key for ligand binding. Phe40 and Phe390 contribute through π–cation and hydrophobic interactions that anchor bromhexine, while Asn394 forms hydrogen bonds, collectively maintaining the ligand’s stable conformation within the binding site. Current evidence indicates that bromhexine inhibits SARS-CoV-2 infection by disrupting the interaction between the viral spike protein and the ACE2 receptor, further supporting its potential as an antiviral agent.

## 2 Materials and methods

### 2.1 Cell lines and reagents

The human embryonic kidney cell lines HEK-293 and HEK-293T were obtained from the American Type Culture Collection (Manassas, VA, United States). HEK-293 cells were cultured in Dulbecco’s Modified Eagle’s Medium (DMEM/F-12) (Invitrogen Life Technologies, Carlsbad, CA, United States) supplemented with 10% (v/v) fetal bovine serum (FBS) (Thermo Fisher Scientific, Waltham, MA, United States) and 1% antibiotics (100 U/mL penicillin and 100 μg/mL streptomycin) (Cytiva HyClone, Thermo Fisher Scientific). The HEK-293T cells, used for generating lentiviral particles, were cultured in high-glucose DMEM (Thermo Fisher Scientific) with penicillin and streptomycin (100 U/mL and 100 μg/mL, respectively), along with 10% (v/v) FBS. Cell lines were maintained at 37 °C in a 5% CO_2_ atmosphere.

A stock solution of 50 mM bromhexine or bromhexine hydrochloride (*N*-cyclo-*N*-methyl-(2-amino-3,5-dibromo-benzyl) amine hydrochloride) (Sigma-Aldrich, St. Louis, MO, United States) was prepared in dimethyl sulfoxide (DMSO; Sigma-Aldrich) and stored at −20 °C. Working concentrations of bromhexine were freshly prepared in cell culture media just before use. The control groups in the respective experiments were treated with an equivalent volume of DMSO. The final DMSO concentration was kept below 0.1%, which is considered safe and non-toxic for cell lines.

### 2.2 Production of lentiviral particles

Lentiviral particles were generated by co-transfecting HEK-293T cells with 3 µg of the vector pLENTI_hACE2_PURO (Addgene plasmid #155295, Watertown, MA, United States), 2 µg of the packaging plasmid psPAX2 (Addgene #12259), and 1 µg of the envelope protein plasmid pCMV-VSV-G (Addgene #8454), using Lipofectamine 2000 (Invitrogen) following the manufacturer’s protocol. After 24 h post-transfection, the culture media was replaced with fresh high-glucose DMEM and kept at 37 °C with 5% CO_2_ for 72 h. The supernatant containing the lentiviral particles was collected and pooled. Cellular debris was removed by filtering through a 0.45 µm filter, and the clarified supernatants were aliquoted and stored at −80 °C until further use.

### 2.3 Generation of a stable HEK-293 cell line expressing human ACE2 protein

To generate the stable cell line, HEK-293 cells were cultured until they reached 60%–70% confluence. Next, HEK-293 cells were transduced with lentiviral vectors encoding hACE2. The culture medium was then replaced with an infection mixture consisting of 4 mL of DMEM/F-12 medium, 1 mL of medium containing lentivirus particles, and 5 µL of polybrene (Sigma-Aldrich). After incubation at 37 °C and 5% CO_2_ for 24 h, the infection mixture was removed and replaced with fresh DMEM/F-12 medium. Three days later, selection was started using 2 μg/mL of puromycin (Thermo Fisher Scientific). Human (hACE2) protein expression in the stable HEK-293 cell line (HEK-293/ACE2) was confirmed through immunoblotting and immunocytochemistry analyses.

### 2.4 Immunocytochemistry

The cellular localization of human ACE2 protein in HEK-293 and HEK-293/ACE2 cells was examined using immunofluorescence. Briefly, cells were seeded on coverslips, fixed with 4% paraformaldehyde (PFA) in 1x phosphate-buffered saline (PBS) for 20 min at room temperature, and permeabilized with 2% bovine serum albumin (BSA) in PBS containing 0.1% Triton X-100 for 30 min. After blocking, cells were incubated overnight at 4 °C with mouse monoclonal primary antibodies against ACE2 (1:50 dilution, sc-390851; Santa Cruz Biotechnology, Dallas, TX, United States). Negative controls underwent the same process but used 1x PBS instead of the primary antibody. Next, cells were incubated at room temperature for 1 h with a goat anti-mouse IgG Alexa Fluor 555-conjugated secondary antibody (A-21422; Invitrogen) at a 1:1000 dilution. DAPI (4′,6-diamidino-2-phenylindole; Thermo Fisher Scientific) was then used to stain cell nuclei at a concentration of 0.1 μg/mL for 5 min. Immunofluorescence images were captured using an Olympus BX53 fluorescence microscope (Olympus Corp., Tokyo, Japan), equipped with an Olympus DP28 camera. Digital images were acquired with Olympus cellSens Entry 3.1.1 (Build 21264) software. Image analysis was performed using ImageJ software version 1.49d (National Institutes of Health, Bethesda, MD, United States). All staining procedures were carried out in triplicate using independent cultures.

### 2.5 Protein extraction and Western blotting

Proteins were extracted from cultured HEK-293 and HEK-293/ACE2 cells with at least 70% confluence. The cells were gently scraped, pelleted by centrifugation at 5000 *g* for 5 min at 4 °C, re-suspended, and lysed in ice-cold RIPA buffer (50 mM Tris-HCl pH 8, 150 mM NaCl, 0.5% sodium deoxycholate, 1% Nonidet-P40 (NP-40), 0.1% sodium dodecyl sulfate (SDS), and 1 mM EDTA) supplemented with 1:100 (v/v) Halt protease and phosphatase inhibitor cocktail (Thermo Fisher Scientific). Protein concentration was measured using a protein assay kit (Pierce BCA Protein Assay Kit, Thermo Fisher Scientific). For Western blot, 30 µg of protein were loaded onto 7% SDS-PAGE gels. Proteins were then transferred to nitrocellulose membranes (Thermo Fisher Scientific) and blocked with PBST buffer (1x phosphate-buffered saline and 0.1% Tween-20) containing 5% non-fat milk overnight at 4 °C. After blocking, the membranes were incubated with primary antibodies against ACE2 (sc-390851, Santa Cruz Biotechnology), and Actin (sc-1615, Santa Cruz Biotechnology) at room temperature for 4 h. Subsequently, membranes were incubated with HRP-conjugated secondary antibodies at room temperature for 2 h, and the antibody-antigen complexes were detected using SuperSignal West Femto Maximum Sensitivity Substrate (Thermo Fisher Scientific). The Omega Lum™ Imaging System (Aplegen, Pleasanton, CA, United States) was used to visualize protein bands. Protein levels in the Western blots were quantified by densitometry, and band density was analyzed using ImageJ software. Analyses were performed across three independent experiments.

### 2.6 MTT cell viability assay

To assess the cytotoxicity of bromhexine, we examined cell viability using the MTT assay at concentrations of 0, 0.01, 0.1, 1, 10, 100, and 250 µM bromhexine for 48 h in HEK-293 cells. To evaluate cell viability in response to bromhexine, 3 × 10^3^ HEK-293 cells/mL were seeded in 96-well plates and cultured for 24 h at 37 °C with 5% CO_2_ in triplicate. Then, medium containing 0.1% DMSO (vehicle), 0, 0.01, 0.1, 1, 10, 100, and 250 µM bromhexine (Sigma-Aldrich) was added to the cultures and incubated for 48 h. After incubation, cells were treated with 10 µL of the MTT (3-[4,5-dimethylthiazol-2-yl]-2,5-diphenyltetrazolium bromide) reagent (Cell Proliferation Kit I (MTT); Roche, Sigma-Aldrich) per well at a final concentration of 0.5 mg/mL and incubated for 4 h. Finally, 100 µL of solubilization buffer was added to the cultures and incubated overnight. Cell viability was measured by reading the absorbance using a microplate reader Sunrise (Tecan, Männedorf, Switzerland) at 570 nm, with a reference reading at 690 nm. Analysis curves were generated at 48 h post-seeding in three independent experiments.

### 2.7 Production of Omicron pseudovirus and other variants

Pseudoviruses expressing the Omicron S constructs were generated through transient co-transfection of HEK-293T cells with 1.5 µg of pcDNA3.3_SARS2_omicron_BA.1 (Addgene #180375) expression vector for the SARS-CoV-2 spike protein, 2 µg of helper plasmid (psPAX2), 1.5 µg of reporter gene (pFUGW-Pol2-ffLuc2-eGFP, Addgene #71394), and 1 µg of pCMV-VSV-G using Lipofectamine 2000 (Invitrogen). After 24 h, the cell media was replaced with fresh high-glucose DMEM and maintained at 37 °C with 5% CO_2_ for 72 h. The SARS-CoV-2 spike pseudoviruses were then filtered through a 0.45 µm filter, aliquoted, and stored at −80 °C. To produce other variants, vectors such as pcDNA3.3_CoV2_501V2 (Addgene #170449, South Africa strain/beta strain), pcDNA3.3_CoV2_B.1.1.7 (Addgene #170451, United Kingdom strain/alpha strain), and pcDNA3.3_SARS2_B.1.617.2 (Addgene #172320, delta strain) were used, following the same protocol.

### 2.8 Omicron pseudovirus infectivity assay

HEK-293/ACE2 cells (5 × 10^4^ cells/well) were seeded on coverslips in 24-well plates and incubated at 37 °C with 5% CO_2_ for 24 h. Cells were pretreated with 1, 10, and 100 µM bromhexine for 2 h, then incubated with pseudoviruses for 48 h. Afterward, the cells were fixed with 4% PFA in 1x PBS for 30 min to inactivate the virus. The fixative was removed, and cells were washed with 1x PBS. Next, nuclei were stained with DAPI at a concentration of 0.1 μg/mL for 5 min at room temperature in the dark. Viral infection was measured by assessing fluorescence levels. Quantitative analysis of GFP fluorescence from microscopy images was performed using ImageJ software. All experiments were conducted at least three times with independent cell cultures.

### 2.9 Luciferase assay

HEK-293/ACE2 cells (3 × 10^3^ cells/mL) were seeded in 96-well plates and cultured in quadruplicate for 24 h at 37 °C with 5% CO_2_. Cells were pretreated with 0, 0.01, 0.1, 1, 10, 100, and 500 µM bromhexine for 2 h, then incubated with pseudoviruses for 48 h. Viral infection was assessed by measuring luciferase activity. Luminescence was detected with the Pierce Renilla-Firefly Luciferase Dual Assay Kit (Thermo Fisher Scientific) according to the manufacturer’s instructions. Luminescence readings were taken at room temperature using an Infinite Lumi microplate reader (Infinite 200 PRO, Tecan Trading AG).

### 2.10 Dose-response (IC_50_) of bromhexine against HEK-293/ACE2 cells

The IC_50_ values of bromhexine against Omicron pseudovirus was determined by pretreating HEK-293/ACE2 cells for 2 h with 0, 0.01, 0.1, 1, 10, 100, and 500 µM bromhexine, followed by viral infection for 48 h. Viral infection was assessed by measuring luciferase activity and quantifying it as described above. The IC_50_ experiments were repeated at least three times with five replicates each. Curves were fitted using a four-parameter logistic function and constructed based on the average parameters from individual experiments.

### 2.11 Effect of bromhexine on infectivity of HEK-293/ACE2 cells

To evaluate the effect of bromhexine on Omicron pseudovirus infection, HEK-293/ACE2 cells (5 × 10^4^ cells/well) were seeded on coverslips in 24-well plates and incubated at 37 °C with 5% CO_2_ for 24 h. The cells were pretreated with 1, 10, and 100 µM bromhexine for 2 h before being exposed to pseudoviruses for 48 h. Viral infection was measured by evaluating fluorescence levels and was quantified as described above. All experiments were performed at least three times using independent cell cultures.

### 2.12 Molecular docking and molecular dynamics simulations

To evaluate how bromhexine interacts with hACE2, the structure of hACE2 bound to the receptor-binding domain (RBD) of the SARS-CoV-2 spike protein (Protein Data Bank, PDB ID: 6LZG) was used as a model (Wang Q. et al., 2020), given its critical role in mediating viral entry through specific interactions at the molecular interface, this complex displays a network of hydrogen bonds, salt bridges, and hydrophobic contacts involving key residues such as Lys417, Tyr453, and Ans501 of the spike protein, as well as Asp30, His34, Tyr41, and Lys353 of hACE2. These interactions collectively stabilize the virus-receptor binding. The high-resolution crystal structure (2.5 Å) offers a reliable framework for *in silico* docking and molecular dynamics simulations to identify potential inhibitors that could block or modulate this interface (Wang Q. et al., 2020).

Protein preparation was carried out using the Protein Preparation Wizard module in Maestro. Hydrogens were added, and protonation states were assigned for a physiological pH of 7.4 (Sastry et al., 2013), followed by energy minimization using the optimized potentials for liquid simulations (OPLS4) force field.

The docking grid box was positioned on the hACE2 region that interacts with the spike RBD, especially around the key residues previously identified as important for molecular recognition. These include Lys31, His34, Tyr41, Lys353, and Asn355, which are essential for the stability of the complex. Additionally, residues Gln24, Ser19, Asp30, Tyr83, Phe28, Leu79, and Met82 contribute both polar interactions (such as hydrogen and ionic bonds) and hydrophobic contacts. This explains the high affinity of SARS-CoV-2 for hACE2 (Wang Q. et al., 2020). Molecular docking was performed using the Standard Precision (SP) mode of Glide (Sándor et al., 2010), generating the top ten binding poses for bromhexine. These conformations were subsequently refined by calculating the binding free energy (ΔG_Bind_) using the Molecular Mechanics combined with the Generalized Born and Surface Area (MM-GBSA) protocol implemented in Prime (Dasmahapatra et al., 2022).

The complex with the most favorable ΔG_Bind_ value was selected for molecular dynamics (MD) simulations. First, a 20 ns equilibration was conducted using Desmond (Dilipkumar et al., 2024) and the OPLS4 force field (Lu et al., 2021), followed by a 500 ns production MD simulation. The system was solvated with single point charge (SPC) water molecules within a periodic boundary box. Counterions (Na^+^ or Cl^−^) were added to neutralize the overall charge, and a final NaCl concentration of 0.15 M was applied to simulate physiological conditions.

The system was relaxed using the default Desmond relaxation protocol and equilibrated under an NPT ensemble (1 atm, 300 K). A spring constant of 10.0 kcal mol^−1^ Å^−2^ was applied to the ligand and protein during 20 ns of MD equilibration. The final frame of the equilibrated system was then used as the starting point for the 500 ns production run. This last 500 ns MD was performed under the same conditions as described above but without any restrictions on either the proteins or the ligand.

### 2.13 Statistical analysis

Data were collected and analyzed using SigmaPlot software version 12.0 (Systat Software Inc., San Jose, CA, United States). Differences between groups were assessed with one- or two-way analysis of variance (ANOVA), followed by *post hoc* Tukey Honestly Significant Difference (HSD) test. A *p*-value <0.05 was considered statistically significant, and all data are presented as mean ± standard error of the mean (SEM).

## 3 Results

### 3.1 ACE2 expression in HEK-293 cells

**FIGURE 1:**
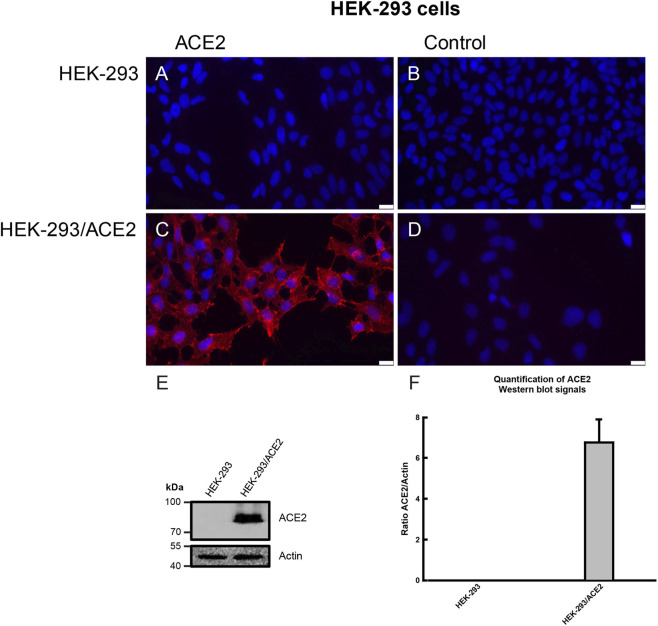
Immunofluorescence and Western blot analysis of ACE2 in HEK-293 and HEK-293/ACE2 cell lines. **(A,C)** Immunofluorescence localization of ACE2 proteins (red fluorescence). **(B,D)** Immunostaining images with primary antibodies omitted (control). All images show cell nuclei stained with DAPI (blue fluorescence). The scale bar represents 20 µm. **(E)** Representative immunoblots for ACE2 (90 kDa) and Actin (42 kDa) as a loading control are shown for HEK-293 cells and the stable cell line overexpressing human ACE2 (HEK-293/ACE2). **(F)** The relative abundance of ACE2 protein levels is expressed as the ratio of ACE2 to Actin band intensities. Data are shown as mean ± SEM from three independent experiments.

HEK-293 cells that do not express angiotensin-converting enzyme 2 (ACE2) cannot be infected by SARS-CoV-2 or spike-pseudotyped viruses (Supplementary Figure S1). To create a suitable cellular model for studying SARS-CoV-2 pseudovirus infection, we developed a stable HEK-293 cell line overexpressing human ACE2 (HEK-293/ACE2) (Figure 1). This line consistently demonstrates high and sustained human ACE2 (hACE2) expression across multiple passages. We confirmed the presence of hACE2 in HEK-293/ACE2 and in HEK-293 cells using immunofluorescence. A positive hACE2 immunoreactive signal was observed only in HEK-293/ACE2 cells (Figure 1C), consistent with the expected membrane localization pattern. This shows that hACE2 is stably expressed and primarily located on the surface of the HEK-293/ACE2 cell line. Positive labeling for hACE2 was not detected when the primary antibody was omitted (controls, Figure 1D). To verify the immunofluorescence results, protein levels of hACE2 were also measured by Western blotting (Figures 1E,F). Immunoblotting of HEK-293/ACE2 cell lysate showed a protein band migrating at approximately 90 kDa, consistent with the predicted molecular size of human ACE2 (Figure 1E). The HEK-293 cell lysate did not show any signal for hACE2 protein (Figure 1E). A densitometric analysis of the relative intensity of the 90 kDa hACE2 band, normalized to Actin, is presented in Figure 1F. Actin was used as a loading and blotting control, with no variation observed in Actin protein levels (Figure 1E). Actin-normalized densitometry values showed significantly higher levels of hACE2 protein in HEK-293/ACE2 cells and were not detected in HEK-293 cells (Figure 1F). Consistent with the immunofluorescence results, hACE2 protein levels were also only detectable in HEK-293/ACE2 cells compared to levels in HEK-293 cells (Figures 1E,F).

### 3.2 Bromhexine decreases the infectivity of Omicron pseudoviruses in HEK-293/ACE2 cells

**FIGURE 2:**
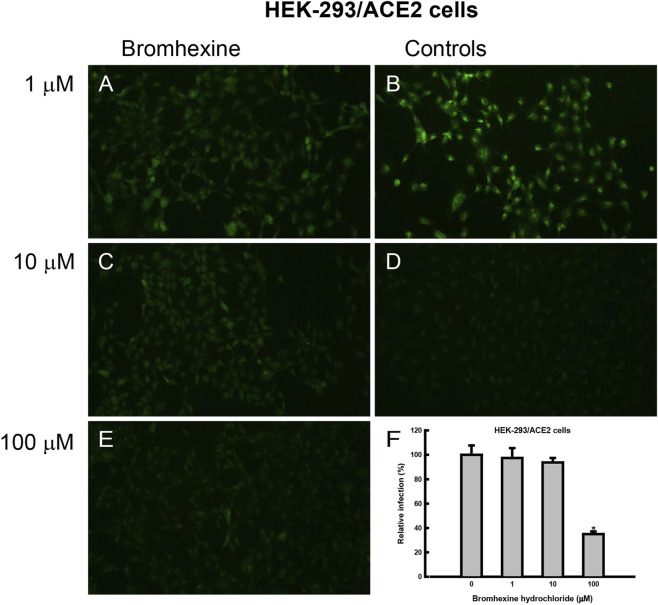
Effect of bromhexine on SARS-CoV-2 Omicron pseudovirus infectivity in HEK-293/ACE2 cells. **(A,C,E)** Representative fluorescence microscopy images of cells infected with Omicron pseudovirus treated with 1, 10, and 100 µM bromhexine, respectively. **(B)** Positive control (Omicron pseudovirus infection without bromhexine). **(D)** Negative control (cells without Omicron pseudovirus infection or bromhexine). **(F)** Quantitative analysis of Omicron pseudovirus infection in HEK-293/ACE2 cells, based on the percentage of GFP-positive cells after infection. GFP expression indicates successful pseudovirus entry. Data are presented as means ± SEM (*n* = 4) from at least three different experiments. An asterisk (*) indicates statistically significant differences (*p* < 0.05) compared to the positive control, determined by one-way ANOVA with *post hoc* Tukey HSD test.

**FIGURE 3:**
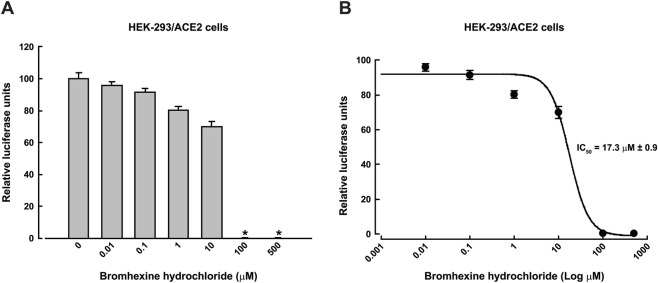
Luciferase activity and IC_50_ determination on HEK-293/ACE2 cells. **(A)** Infectivity of Omicron pseudoviruses was assessed by measuring luciferase activity in relative luminescence units (RLUs) after infecting cells with the viruses. Data are shown as means ± SEM (*n* = 4). An asterisk (*) indicates statistically significant differences (*p* < 0.05) determined by one-way ANOVA with *post hoc* Tukey HSD test. **(B)** Dose-response curve used to determine the half-maximal inhibitory concentration (IC_50_) of bromhexine in HEK-293/ACE2 cells infected with Omicron pseudovirus, with an IC_50_ of 17.3 ± 0.9 µM. Results are presented as means ± SEM (*n* = 4). Curves are fitted to a 4-parameter logistic model and generated using the average of fitted parameters from individual experiments.

To evaluate bromhexine’s cytotoxicity, we measured cell viability using the MTT assay at concentrations of 0, 0.01, 0.1, 1, 10, 100, and 250 µM bromhexine for 48 h in HEK-293 cells (Supplementary Figure S2). We then assessed the effect of bromhexine on cell infection at concentrations from 0 to 100 µM over 48 h in HEK-293/ACE2 cells infected with Omicron pseudoviruses (Figures 2A–E). The level of infection with Omicron pseudoviruses, which include the reporter genes GFP and luciferase, could be easily detected in cells using fluorescence microscopy and by measuring luciferase activity in HEK-293/ACE2 cells incubated with bromhexine (Figures 2A–E). Infectivity was quantified by calculating the percentage of GFP-positive cells, showing approximately 60% suppression of infection at a concentration of 100 µM bromhexine (Figure 2F). Additionally, a more quantitative approach involves measuring luciferase activity. Luciferase assays confirmed the inhibitory effect of bromhexine, as shown in Figures 3A,B. This inhibition by bromhexine yielded an IC_50_ of 17.3 ± 0.9 μM (Figure 3B).

### 3.3 Bromhexine decreases infectivity of alpha, beta, and delta pseudoviruses in HEK-293/ACE2 cells

**FIGURE 4:**
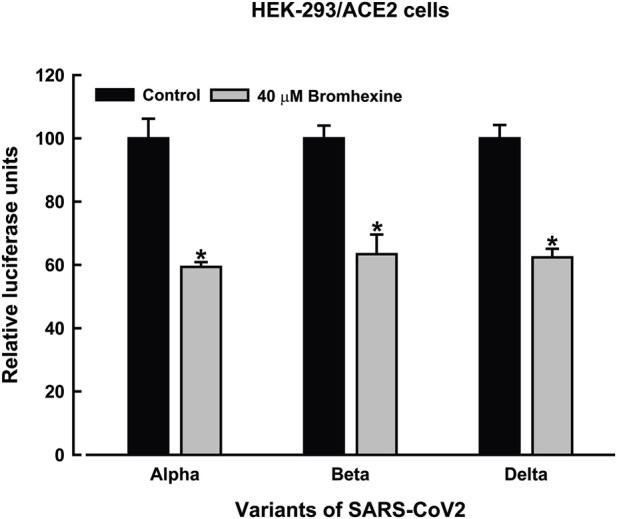
Reduction in infectivity of SARS-CoV-2 pseudovirus variants in HEK-293/ACE2 cells treated with bromhexine. Infectivity of pseudoviruses representing Alpha, Beta, and Delta SARS-CoV-2 variants was measured by luciferase activity, expressed in relative luminescence units (RLUs), 48 h after treatment with 40 μM bromhexine or a vehicle (control). The cells were infected with pseudoviruses engineered to express each variant. Data are shown as means ± SEM (*n* = 4). An asterisk (*) indicates statistically significant differences (*p* < 0.05) compared to the control group, determined by two-way ANOVA followed by *post hoc* Tukey HSD test.

**FIGURE 5:**
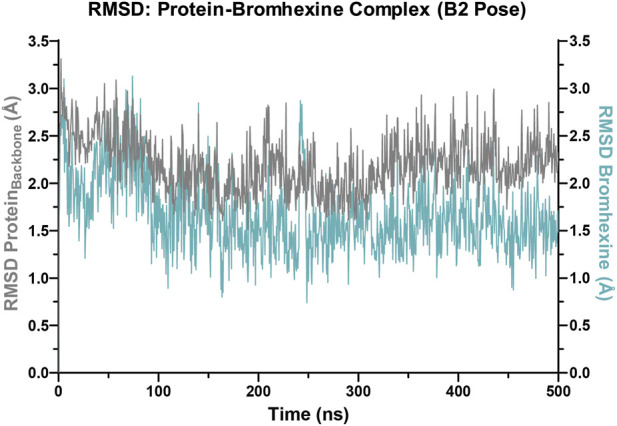
Illustration of the root mean square deviation (RMSD) profiles of the hACE2-bromhexine complex over a 500 ns molecular dynamics simulation. The plot shows the RMSD progress of the hACE2 backbone (gray, left Y-axis) and bromhexine (light blue, right Y-axis) relative to their initial positions during the simulation. The protein backbone remains in a stable conformation with minimal fluctuations throughout the simulation. Bromhexine maintained a sustained binding within the pocket, without significant displacements over the simulation.

Next, we examined the impact of bromhexine on HEK-293/ACE2 cells infected with various pseudovirus variants. Experiments used 40 µM bromhexine (the upper limit of the IC_50_ range), and infection was assessed after 48 h of incubation (Figure 4). Figure 5 shows the infection of HEK-293/ACE2 cells with different pseudovirus variants (Alpha, Beta, and Delta). Our results indicated that treatment with 40 µM bromhexine resulted in roughly a 40% decrease in infection across all three variants (Figure 4).

### 3.4 The bromhexine exhibits an energetically stable interaction with the hACE2 receptor

To study the interaction between bromhexine and the hACE2 receptor, we conducted molecular docking using the crystallographic structure of the SARS-CoV-2 spike receptor-binding domain (RBD) complexed with hACE2 (PDB ID: 6LZG). The docking was focused on the interface between the spike RBD and hACE2 to determine if bromhexine could potentially disrupt this essential protein–protein interaction. The results were then rescored with the MM-GBSA method to better estimate the binding free energy (ΔG_Bind_).

The docking results, summarized in Table 1, include both the docking scores and the MM-GBSA ΔG_Bind_ values for the bromhexine–hACE2-RBD complex. Lower docking scores suggest stronger predicted binding affinity, while the MM-GBSA ΔG_Bind_ values provide a thermodynamic perspective by incorporating solvation contributions. Bromhexine exhibited favorable binding characteristics, with a notable docking score and a significantly negative ΔG_Bind_ (−53.01 kcal/mol), indicating a stable and potentially inhibitory interaction with the hACE2 binding site.

**TABLE 1:** Docking score and MM-GBSA binding free energy (ΔG_Bind_) values for the top-10 bromhexine docking poses with the ACE2-RBD complex.

Bromhexine pose	Docking score	MM-GBSA ΔG_Bind_ (kcal/mol)
B1	−4.284	−46.52
B2	−4.205	−53.01
B3	−4.096	−25.53
B4	−3.563	−34.01
B5	−3.520	−36.15
B6	−3.422	−26.99
B7	−3.175	−32.22
B8	−2.899	−33.56
B9	−2.684	−33.35
B10	−2.489	−23.61

MM-GBSA, molecular mechanics-generalized born surface area; ΔG_Bind_, binding free energy. Poses were ranked by docking score, and binding energies were calculated using the MM-GBSA, method. The pose with the most favorable ΔG_Bind_ (B2) was selected for molecular dynamics simulations.

Next, a molecular dynamics (MD) simulation was performed to examine the interaction between bromhexine in its B2 pose—refer to Table 1—and the protein structure from the crystallographic complex with hACE2-RBD (PDB ID: 6LZG). This pose was chosen because it demonstrated the most favorable ΔG_Bind_ according to the MM-GBSA analysis (Table 1). The simulation was performed for 500 ns, allowing for the evaluation of the complex’s temporal evolution, with special emphasis on its structural stability and the conformational behavior of the ligand.

To assess the temporal stability of the bromhexine–ACE2 interaction, we tracked the root mean square deviation (RMSD) of both the protein backbone and the ligand’s position within the binding site during the 500 ns MD simulation. As shown in Figure 5, the protein backbone RMSD of ACE2 (gray line) stayed around 2.0–2.5 Å, indicating a stable overall conformation of the protein over the simulation period. Meanwhile, bromhexine (light blue line) showed lower RMSD values near 1.5 Å, with no signs of significant fluctuations or dissociation events within the binding pocket. These findings suggest that bromhexine maintains a consistent and stable interaction with the ligand and the hACE2 binding pocket, supporting the docking and binding energy data.

This molecular dynamics analysis provides additional insights beyond docking and MM-GBSA results by emphasizing the dynamic behavior and structural stability of the ligand–protein complex under simulated physiological conditions. It supports the idea of a stable ligand–receptor interaction and the proposed inhibitory mechanism of bromhexine at the ACE2 interface.

### 3.5 The residues Phe40, Phe390, and Asn394 facilitate bromhexine binding through π–cation and hydrophobic interactions

**FIGURE 6:**
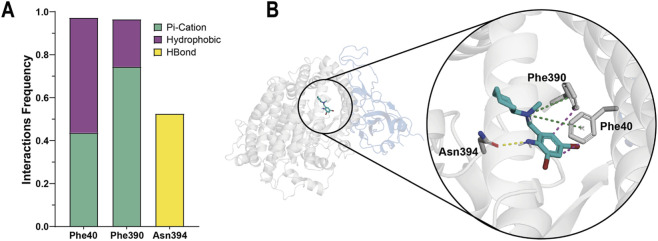
Molecular interactions between bromhexine and key hACE2 residues. **(A)** Interaction frequency plot of Phe40, Phe390, and Asn394 residues over 500 ns of MD simulations. Frequencies indicate the proportion of simulation time during which each interaction type was maintained (π–cation, hydrophobic, or hydrogen bond). Only residues with an interaction frequency of at least 0.2 during the 500 ns simulation are shown. **(B)** Structural representation of bromhexine in complex with hACE2 at the 500 ns snapshot. The spike RBD (blue) and hACE2 (white) are depicted as ribbons, with bromhexine shown in stick format. Dashed lines indicate π–π-cation (green), hydrophobic (magenta), and hydrogen bond (yellow) interactions with the specified residues.

To better understand how bromhexine binds to hACE2 and to identify the roles of key residues, we conducted a detailed residue–ligand interaction analysis during a 500 ns MD simulation of the complex. The focus was on residues interacting with the ligand for more than 20% of the simulation time, which are likely important for bromhexine binding. These hACE2 residues are crucial because of their unique chemical properties and exact location within the interface. As shown in Figure 6A, Phe40 and Phe390 form recurring π–cationic and hydrophobic interactions with bromhexine, helping to anchor the ligand within the binding pocket. Additionally, the Asn394 residue creates stabilizing hydrogen bonds, supporting the ligand’s stable structure and strong affinity. These interaction profiles were visualized in a 3D snapshot of the complex at 500 ns (Figure 6B), highlighting the ligand’s orientation, key contacts, and the spatial arrangement of important residues surrounding bromhexine in the ACE2 binding pocket. The stability of these interactions align with the RMSD analysis, confirming the formation of a stable and specific ligand–receptor binding mode.

Our findings align with existing literature, as *in vitro* studies have demonstrated that ambroxol (a metabolite of bromhexine) interacts with the hACE2 receptor. This interaction may disrupt the spike glycoprotein of SARS-CoV-2 from binding or promote surfactant secretion in alveolar cells, thereby reducing viral entry (Kehinde et al., 2022).

## 4 Discussion

In this study, we demonstrate that bromhexine hydrochloride significantly reduces SARS-CoV-2 Omicron pseudovirus infection in a stable HEK-293/ACE2 cell line. Confirmation of human ACE2 expression through immunofluorescence and Western blot verifies the reliability of our *in vitro* model for studying viral entry processes, consistent with previous research highlighting the critical role of ACE2 in SARS-CoV-2 infection (Li et al., 2003; Zhu et al., 2020; Jackson et al., 2022).

Bromhexine treatment caused a dose-dependent decrease in pseudoviral infectivity, with an IC_50_ of 17.3 ± 0.9 µM. Importantly, bromhexine showed consistent antiviral effects against additional SARS-CoV-2 variants, including Alpha, Beta, and Delta, achieving approximately 40% infection suppression at 40 µM. These findings suggest that bromhexine has broad-spectrum inhibitory potential against emerging viral variants.

Bromhexine is known as a potent and selective inhibitor of the transmembrane serine protease 2 (TMPRSS2) (Lucas et al., 2014). Different reports indicating that bromhexine, by targeting TMPRSS2, can interfere with viral entry processes (Shen et al., 2017; Gil et al., 2020; Hoffmann et al., 2020b). Mechanistically, the observed antiviral effects may be due to the known bromhexine inhibition of TMPRSS2, a host protease vital for activation of the spike protein on the cell surface, which produces conformational changes in the S1–ACE2 complex required for fusion of the viral membrane with the plasma membrane (Hoffmann et al., 2020b; Zhu et al., 2020).

Our model for studying SARS-CoV-2 pseudovirus infection was a stable HEK-293 cell line overexpressing human ACE2 (HEK-293/ACE2) in the absence of TMPRSS2. We demonstrated that TMPRSS2 expression in HEK-293/ACE2 cells was not required for SARS-CoV-2 pseudovirus infection. These results imply that proteolytic activation by TMPRSS2 may not be a limiting factor for SARS-CoV-2 infection. This suggest that the virus might use alternative pathways for cellular entry (Hoffmann et al., 2020b), where SARS-CoV-2 binding to ACE2, in the absence of TMPRSS2, initiates endosomal entry of the viral particle.

Additionally, molecular interaction analyses indicate a possible direct disruption of spike-ACE2 binding (Yan et al., 2020; Zhu et al., 2020; Xiao et al., 2021). This dual mechanism—blocking both host protease activity and virus–host receptor interaction—may explain bromhexine’s strong effectiveness across various variants.

Although ACE2 and TMPRSS2 are co-expressed in a limited number of tissues (Hoffmann et al., 2020b), understanding the structural and regulatory role of ACE2 and TMPRSS2 could reveal important insights into differential susceptibility to SARS-CoV-2 infections and novel targets therapeutic purposes.

Nonetheless, pseudovirus models are limited to assessing viral entry and do not cover later stages of the viral replication cycle. Therefore, verifying these results with authentic SARS-CoV-2 isolates in more complex biological systems, such as animal models and clinical settings, is crucial. Additionally, translating effective *in vitro* concentrations to therapeutic doses in humans necessitates careful pharmacokinetic and toxicological evaluation.

Our binding free energy analysis is consistent with the value of ΔG_Bind_ (−46.354 kcal/mol), reported in 2022, for MM-GBSA calculations of the bromhexine–hACE2 complex (Kehinde et al., 2022). Although its docking score was not the lowest in the group (−4.205 kcal/mol), post-docking energy refinement shows a more favorable conformational adjustment for interacting with key residues, such as Lys353, His34, and Tyr41, which are identified as critical for viral recognition (Pirolli et al., 2023). Therefore, bromhexine seems to bind within the extracellular peptidase domain of hACE2, near key residues Glu37, His34, and Tyr41. The pattern of hydrogen bonds and hydrophobic interactions supports the idea that bromhexine might influence viral entry not only by inhibiting TMPRSS2 but also by directly attaching to hACE2.

Finally, molecular dynamics (MD) simulations provided additional evidence, showing that bromhexine interactions with hACE2—particularly hydrophobic contacts with Phe40 and Phe390, and the hydrogen bond with Asn394—remain stable during the 500 ns trajectory. These contacts are consistent with the RMSD data and reinforce the idea of a sustained and specific binding mode.

## 5 Conclusion

Our findings identify bromhexine hydrochloride as an effective inhibitor of SARS-CoV-2 pseudovirus entry across various variants of concern. This underscores bromhexine’s potential as a repurposed treatment for COVID-19, especially in the early stages of infection. Future studies should focus on testing bromhexine’s antiviral effects against live virus, evaluating its effectiveness *in vivo*, and exploring possible synergy with other antiviral drugs. Additionally, pinpointing key residues at the spike–hACE2 interface offers new opportunities for designing advanced viral entry inhibitors, enhancing our defenses against both current and future coronaviruses. Overall, these results emphasize the potential of bromhexine as a cost-effective, widely accessible therapeutic option, supporting global efforts to repurpose approved drugs for rapid deployment in post-pandemic scenarios.

## Data Availability

The original contributions presented in the study are included in the article/Supplementary Material, further inquiries can be directed to the corresponding author.
