# Supplementary material for: Bromhexine inhibits SARS-CoV-2 Omicron and variant pseudovirus infection via ACE2-targeted mechanisms
Source: Front Pharmacol. 2026 Jan 12;16:1745277. doi: 10.3389/fphar.2025.1745277 (PMC12832721; doi:10.3389/fphar.2025.1745277)
Supplement: Supplementary file 1 [file Table1.docx]

**Supplementary figure captions**

**Fig. 1S. Relative expression analysis of *ACE2* and *TMPRSS2* in HEK-293 and HEK-293/ACE2 cells.** Expression levels of *ACE2* and *TMPRSS2* mRNA in HEK-293 cells were measured using quantitative real-time PCR (Supplementary methods). Gene expression was normalized to the housekeeping gene *Homo sapiens* ribosomal protein L19 (RPL19) with the ΔCt method. Data are presented as mean ± SEM (*n* = 3) from two separate experiments.

**Fig. S2. Effect of bromhexine hydrochloride on HEK-293 cell viability.** HEK-293 cells were treated for 48 hours with various concentrations of bromhexine (0-250 µM) or DMSO, and cell viability was measured using the MTT assay after treatment. Values were normalized to the vehicle control (DMSO-treated cells), set at 100 % viability. The percentage of viable cells is shown as means ± SEM (*n* = 4) from at least three different experiments. An asterisk (*) indicates statistically significant differences (*p* < 0.05) compared to the DMSO control group, based on one-way ANOVA with post-hoc Tukey HSD-test. Double asterisks (**) indicate highly statistically significant differences (*p* < 0.001) and denote complete loss of viability at 250 µM bromhexine.
