# Supplementary material for: Bromhexine inhibits SARS-CoV-2 Omicron and variant pseudovirus infection via ACE2-targeted mechanisms
Source: Front Pharmacol. 2026 Jan 12;16:1745277. doi: 10.3389/fphar.2025.1745277 (PMC12832721; doi:10.3389/fphar.2025.1745277)
Supplement: Supplementary file 4 [file Table2.docx]

**Supplementary method**

**Extraction and quantification of mRNA using real-time PCR**

Total RNA was isolated from HEK-293 and HEK-293/ACE2 cells using TRIzol Reagent (Invitrogen, Thermo Fisher Scientific), followed by an additional DNase treatment with the TURBO DNA-free kit (Life Technologies). First-strand cDNA was synthesized from 1 µg of RNA using oligo(dT) primers and the RevertAid H Minus First Strand cDNA Synthesis Kit (Thermo Fisher Scientific) at 42 °C for 60 minutes. The resulting cDNA served as a template for PCR amplification of the *ACE2*, *TMPRSS2*, and *RPL19* genes with specific primers (Supplementary Table S1).

The real-time PCR reactions included 12.5 µL of 2x Brilliant II SYBR Green QPCR Master Mix (Agilent Technologies, Santa Clara, CA, USA), 600 nM of each primer, a final reference dye concentration of 30 nM, and 100 ng of cDNA template. The cycling program started with an initial step at 95 °C for 10 minutes, followed by 40 cycles at 95 °C for 30 seconds, 60 °C for 1 minute, and 72 °C for 30 seconds. A final step at 95 °C for 1 minute was performed, followed by melting curve analysis from 55 °C to 95 °C at 0.2 °C/s increments. These assays were run in triplicate on a Stratagene Mx3000P real-time thermal cycler and analyzed with MxPro qPCR software (Agilent Technologies). Primer pairs were tested for efficiency, with only those achieving 90–100 % efficiency selected. The specificity of the PCR products was confirmed through gel electrophoresis and melting curve analyses. Gene expression levels were normalized to the housekeeping gene ribosomal protein L19 (RPL19).

| **Table S1** |  |  |  |  |  |
| --- | --- | --- | --- | --- | --- |
| Primer sets used for quantitative real time RT-PCR analysis. | | | | | |
| **Gene** | **Common name** | **GenBank Accession** | **Abbreviation** | **Primer pair, sense (5’–3’)** | **Product size (bp)** |
| *ACE2* | ACE2 | AY623811 | qHsACE2_F1 | 5’-CAC GAT TGT TGG GAC TCT GCC ATT T-3’ | 131 |
|  |  |  | qHsACE2_R1 | 5’-CCA CCC CAA CTA TCT CTC GCT TCA-3’ |  |
| *TMPRSS2* | TMPRSS2 | AF123453 | qHsTMPRSS2_F1 | 5’-CCC TCT AAC TGG TGT GAT GGC GTG-3’ | 85 |
|  |  |  | qHsTMPRSS2_R1 | 5’-TGA AGT TTG GTC CGT AGA GGC GAA-3’ |  |
| *RPL19* | L19 | NM_000981 | qHsRPL19_F | 5’-CAT CCG CAA GCC TGT GAC G-3’ | 133 |
|  |  |  | qHsRPL19_R | 5’-TGT GAC CTT CTC TGG CAT TCG-3’ |  |
| ACE2, Angiotensin-converting enzyme 2; TMPRSS2, Transmembrane serine protease 2; RPL19, ribosomal protein L19. | | | | | |
